# Impact of the COVID-19 pandemic on continuity of care: a case of mother and son admitted because of psychotic decompensation

**DOI:** 10.1192/j.eurpsy.2026.11015

**Published:** 2026-07-31

**Authors:** I. Samardžić Ilić, M. Ćurković

**Affiliations:** 1 Department of Rehabilitation and Community Support, University Psychiatric Hospital “Vrapče”; 2 University of Zagreb, School of Medicine, Zagreb, Croatia

## Abstract

**Introduction:**

The COVID-19 pandemic disrupted psychiatric care worldwide. Restrictions on mobility, reduced face-to-face visits and sole reliance on telepsychiatry increased the risk of relapse in vulnerable populations such as patients with schizophrenia. Social isolation also placed a significant burden on family caregivers, in some cases contributing to secondary psychopathology.

**Objectives:**

We aim to present a case of a patient with chronic disorganized schizophrenia who discontinued regular follow-up during the COVID-19 pandemic, and his mother who subsequently developed a delusional disorder, with suspicion that her delusions may represent *folie à deux.* This case illustrates the long-term consequences of disrupted psychiatric care and social isolation.

**Methods:**

Clinical case report based on hospital admission records, psychiatric assessment, and review of available outpatient documentation.

**Results:**

A 45-year-old male with chronic disorganized schizophrenia, treated since 2007, discontinued regular psychiatric follow-up after a telepsychiatric consultation in 2020, during which his mother reported that they “mainly stayed at home due to the pandemic.” His last in-person visit was in mid-2024; subsequently, only prescriptions were renewed by primary care. In September 2025, both he and his 76-year-old mother were admitted to our clinic. **Patient (son):** presented with disorganized thought processes, persecutory delusions, weight loss, and scabies. He reported not attending controls because his mother did not want to remain alone. Both he and his mother described people entering their flat at night as well as seeing “flying saucers.” **Patient (mother):** first psychiatric admission. Diagnosed with F06.2 Organic delusional disorder, with clinical suspicion of a dependent personality disorder. She was malnourished, cachectic, hypertensive, and had scabies. Her delusional content overlapped with her son’s experiences, raising suspicion that her psychosis could represent *folie à deux.* Both required inpatient stabilization, psychopharmacological treatment, somatic care including nutritional support, and treatment of scabies.

**Conclusions:**

This case highlights how pandemic-related restrictions and sole reliance on not yet completely established telepsychiatry without in-person assessments may lead to loss of follow-up and severe relapse in chronic psychotic disorders. Furthermore, prolonged social isolation and caregiver burden may precipitate secondary psychotic illness in family members. Future care models should ensure hybrid follow-up systems, integrating telepsychiatry with scheduled in-person evaluations, and include monitoring of vulnerable family dynamics.

**Disclosure of Interest:**

None Declared

